# Making Common Fund data more findable: catalyzing a data ecosystem

**DOI:** 10.1093/gigascience/giac105

**Published:** 2022-11-21

**Authors:** Amanda L Charbonneau, Arthur Brady, Karl Czajkowski, Jain Aluvathingal, Saranya Canchi, Robert Carter, Kyle Chard, Daniel J B Clarke, Jonathan Crabtree, Heather H Creasy, Mike D'Arcy, Victor Felix, Michelle Giglio, Alicia Gingrich, Rayna M Harris, Theresa K Hodges, Olukemi Ifeonu, Minji Jeon, Eryk Kropiwnicki, Marisa C W Lim, R Lee Liming, Jessica Lumian, Anup A Mahurkar, Meisha Mandal, James B Munro, Suvarna Nadendla, Rudyard Richter, Cia Romano, Philippe Rocca-Serra, Michael Schor, Robert E Schuler, Hongsuda Tangmunarunkit, Alex Waldrop, Cris Williams, Karen Word, Susanna-Assunta Sansone, Avi Ma'ayan, Rick Wagner, Ian Foster, Carl Kesselman, C Titus Brown, Owen White

**Affiliations:** Population Health and Reproduction, UC Davis, Davis, CA 95616, USA; University of Maryland Institute for Genome Sciences, University of Maryland School of Medicine, MD 21201, USA; University of Southern California Information Sciences Institute, CA 90292, USA; University of Maryland Institute for Genome Sciences, University of Maryland School of Medicine, MD 21201, USA; Population Health and Reproduction, UC Davis, Davis, CA 95616, USA; University of Maryland Institute for Genome Sciences, University of Maryland School of Medicine, MD 21201, USA; Division of Decision and Information Sciences, University of Chicago and Argonne National Laboratory, Chicago, IL 60637, USA; Department of Pharmacological Sciences, Mount Sinai Center for Bioinformatics, Icahn School of Medicine at Mount Sinai, New York, NY 10029, USA; University of Maryland Institute for Genome Sciences, University of Maryland School of Medicine, MD 21201, USA; University of Maryland Institute for Genome Sciences, University of Maryland School of Medicine, MD 21201, USA; University of Southern California Information Sciences Institute, CA 90292, USA; University of Maryland Institute for Genome Sciences, University of Maryland School of Medicine, MD 21201, USA; University of Maryland Institute for Genome Sciences, University of Maryland School of Medicine, MD 21201, USA; UC Davis Medical Center, Davis, CA 95616, USA; Population Health and Reproduction, UC Davis, Davis, CA 95616, USA; University of Maryland Institute for Genome Sciences, University of Maryland School of Medicine, MD 21201, USA; University of Maryland Institute for Genome Sciences, University of Maryland School of Medicine, MD 21201, USA; Department of Pharmacological Sciences, Mount Sinai Center for Bioinformatics, Icahn School of Medicine at Mount Sinai, New York, NY 10029, USA; Department of Pharmacological Sciences, Mount Sinai Center for Bioinformatics, Icahn School of Medicine at Mount Sinai, New York, NY 10029, USA; Population Health and Reproduction, UC Davis, Davis, CA 95616, USA; Division of Decision and Information Sciences, University of Chicago and Argonne National Laboratory, Chicago, IL 60637, USA; Population Health and Reproduction, UC Davis, Davis, CA 95616, USA; University of Maryland Institute for Genome Sciences, University of Maryland School of Medicine, MD 21201, USA; RTI International, Research Triangle Park 27709-2194, USA; University of Maryland Institute for Genome Sciences, University of Maryland School of Medicine, MD 21201, USA; University of Maryland Institute for Genome Sciences, University of Maryland School of Medicine, MD 21201, USA; Division of Decision and Information Sciences, University of Chicago and Argonne National Laboratory, Chicago, IL 60637, USA; University of Southern California Information Sciences Institute, CA 90292, USA; Interface Guru, Tuscon 85701, USA; Oxford e-Research Centre, Department of Engineering Science, University of Oxford, Oxford OX1 3QG, UK; University of Maryland Institute for Genome Sciences, University of Maryland School of Medicine, MD 21201, USA; University of Southern California Information Sciences Institute, CA 90292, USA; University of Southern California Information Sciences Institute, CA 90292, USA; RTI International, Research Triangle Park 27709-2194, USA; University of Southern California Information Sciences Institute, CA 90292, USA; The Carpentries, Oakland 94607, USA; Oxford e-Research Centre, Department of Engineering Science, University of Oxford, Oxford OX1 3QG, UK; Department of Pharmacological Sciences, Mount Sinai Center for Bioinformatics, Icahn School of Medicine at Mount Sinai, New York, NY 10029, USA; University of California San Diego, San Diego, CA 92093, USA; Division of Decision and Information Sciences, University of Chicago and Argonne National Laboratory, Chicago, IL 60637, USA; University of Southern California Information Sciences Institute, CA 90292, USA; Population Health and Reproduction, UC Davis, Davis, CA 95616, USA; University of Maryland Institute for Genome Sciences, University of Maryland School of Medicine, MD 21201, USA

## Abstract

The Common Fund Data Ecosystem (CFDE) has created a flexible system of data federation that enables researchers to discover datasets from across the US National Institutes of Health Common Fund without requiring that data owners move, reformat, or rehost those data. This system is centered on a catalog that integrates detailed descriptions of biomedical datasets from individual Common Fund Programs’ Data Coordination Centers (DCCs) into a uniform metadata model that can then be indexed and searched from a centralized portal. This Crosscut Metadata Model (C2M2) supports the wide variety of data types and metadata terms used by individual DCCs and can readily describe nearly all forms of biomedical research data. We detail its use to ingest and index data from 11 DCCs.

## Introduction

Findability of existing data in biomedical research is important for data reuse. Reusing data can increase the speed of scientific discovery, as well as allow researchers to generate hypotheses [1–3]. However, these benefits are highly dependent on the (f)indability, (a)ccessibility, (i)nteroperability, and (r)eusability (FAIRness) [4] of individual datasets, with findability by interested researchers being the first essential step. Improving reuse of datasets is increasingly a priority for both scientists and funding agencies [5–7], and there are many data publishing and scientific data repositories that are designed to facilitate targeted search across a broad spectrum of data types [8]. However, researchers still often find it difficult to navigate the many available data repositories or to predict which ones might hold data that are useful to them [9]. Further, researchers browsing a data repository typically rely on topical information to determine if it contains relevant data [10–12], and because most large data repositories typically do not impose constraints on terms used to describe datasets, they are not always well suited to effective searching or browsing [8].

The US National Institutes of Health (NIH) Common Fund (CF) was created in 2006 to fund biomedical research efforts that did not fit into the funding remit of any one NIH institute or center, with the intention of generating unique and catalytic research methods and datasets to fuel future innovation. Nearly 15 years later, more than 50 CF programs have been funded and have created large, diverse collections of genomic, transcriptomic, proteomic, metagenomic, and imaging assets. These data are deep, derived from hundreds of studies, with samples collected from thousands of human subjects, cell lines, organoids, and animal models. Each CF program has a Data Coordination Center (DCC), which facilitates program data storage in repositories or local data centers for public use; most also host curated, derived datasets at program-specific data portals to enable easy use by biomedical researchers. For example, the Genotype-Tissue Expression (GTEx) project [13] offers sophisticated search and contextual display of curated gene expression data, tissue characteristics, and quantitative trait loci (QTLs) at their web portal. The GTEx data portal sees over 15,000 users a month [14] and has enabled hundreds of published studies in which the GTEx data were reused by the global research community.

Although the Common Fund was created to catalyze cross-cutting research and create reusable datasets for biomedical research [15], the many datasets created by different CF programs are neither located in a single repository nor accessible via a common interface. This is an artifact of the funding model: as of 2019, individual programs were isolated, with few connections between active projects and few incentives or opportunities to integrate them [16]. Instead, each program created and managed its own solution for metadata, storage, and research access. Ever-increasing proliferation of access interfaces and storage methods is especially problematic for biomedical researchers, who show a strong interest in reusing data and are the target user base for the Common Fund but who cannot generally maintain expertise in effectively using multiple custom data repositories [6]. The Common Fund Data Ecosystem (CFDE) was established in 2019 both to flatten the siloed nature of CF data and to mitigate the downstream impact such verticalization has had on data reuse.

Before the CFDE, there was little or no contact between Common Fund programs, let alone active collaboration, and with no integration across portals, it was challenging for a researcher to navigate across Common Fund resources. This independence and isolation of different programs has benefits, giving each program freedom to tailor their data-gathering, access interfaces, and infrastructure to answer domain-specific questions and to respond nimbly to changes in mission goals. This independence has, however, also impeded data integration around common data types. Even the seemingly simple task of finding what data are available is hindered by differences in nomenclature and distribution across dozens of programs.

Comparing data across programs is particularly challenging: each program's data portal provides a curated experience of analyzed data that usually does not support comparison with data from other sources. Moreover, many data can only be meaningfully compared to other data processed by the same methods, and because each Common Fund program operates independently, data are stored, labeled, analyzed, curated, and maintained in incompatible ways. Researchers interested in combining data across CF programs must therefore not only find the datasets but also then harmonize across idiosyncratic vocabularies, file types, data structures, and processing methods. Reusing Common Fund data for new cross-cutting scientific analyses requires expertise in working with large files, accessing data in the cloud, harmonization, and data transformation—all before any actual analysis can begin. Each of these processes typically *individually* represents a significant challenge for biomedical researchers and clinicians, motivating labs to hire dedicated bioinformaticians (at considerable cost) to manage them. Confronting *all* of these data-munging operations—necessary before researchers can even begin integrative analysis—often proves prohibitive, truncating or outright eliminating potential investigations.

To make Common Fund data more findable, the CFDE has created a flexible system of data federation that enables users to discover datasets from across the CF at a centralized portal [23] without requiring CF programs to move, reformat, or rehost their data, similar to the federation strategy of the Research Data Alliance [18], the Australian Research Data Commons [19], and the Earth System Grid Federation [20]. The CFDE uses a sociotechnical federation system that combines proven, explicitly community-driven approaches [18, 21, 22] with a model-driven catalog that integrates detailed descriptions of datasets submitted by individual programs’ DCCs into a shared metadata structure that is then indexed and made searchable via a centralized portal.

The sociotechnical framework of the CFDE is a self-sustaining community that both harmonizes existing research metadata and also develops standards with which newly funded programs can guarantee interoperability with the existing informatics ecosystem. Governance of the CFDE includes frequent “cross-pollination” networking events; publication of Requests for Comment (RFC) documents managing community input on metadata standards; extensive documentation of use cases, support infrastructure, and the Crosscut Metadata Model (C2M2); and direct engagement with individual programs to assist and deepen ecosystem participation. In addition, working groups have been formed to guide best practices in areas such as gene- and variant-centric knowledge representation, clinical metadata, shared ontologies for scientific terms, and technical integration strategies. This community effort ultimately manifests as the CFDE portal, a single user-friendly search interface for the Common Fund where all data are comprehensively searchable thanks to harmonization using a common model. This uniform C2M2 supports the wide variety of dataset types, vocabularies, and descriptive metadata used by individual CF DCCs. The C2M2 is designed to be easily extensible to accommodate new DCC-driven use cases and data types: new features are established by ongoing DCC participation in the ecosystem and its working groups, and also by the experiences of DCCs newly joining the CFDE working to integrate existing research data.

The primary user interface for the CFDE's metadata catalog is a web-based portal [23] that supports multifaceted search across a wide variety of datasets using controlled vocabularies describing metadata like anatomy, taxonomy, clinical metadata, assay types, and technical information about data files. This style of search supports common researcher use cases [10, 11], offering custom data filtration of user-selected topical information to rapidly discover datasets that would otherwise require idiosyncratic targeted searches across multiple databases. Findability encompasses more than just searching: it includes the user's experience of interacting with the search interface and their understanding of how to use it to find relevant results. We work closely with a professional usability testing team to ensure that our portal meets user needs.

In this article, we describe the motivation for the C2M2, detail the current C2M2, and discuss the portal that serves integrated metadata from across multiple CF programs. We also describe the strategy that guides C2M2 development and the processes by which the C2M2 evolves.

## Results

### Common Fund data cannot be found by uniform Internet search terms

A biomedical researcher interested in finding Common Fund RNA sequencing (RNA-seq) datasets created from human blood samples should, in theory, be able to find relevant data from at least 5 programs: GTEx, the Gabriella Miller Kids First Pediatric Research Program (GMKF), the Human Microbiome Project (HMP), Extracellular RNA (ExRNA), and the Library of Integrated Network-Based Cellular Signatures (LINCS). Each of these programs hosts their data on a public website; they also offer informational websites about their work, and so one would expect RNA-seq datasets from all of them to be easily findable. Not so: a Google search for “human blood RNAseq 'common fund'” returns 20,500 results, all but 55 of which are omitted by Google as they are “very similar to the 55 already displayed” [24]. These 55 results contain references to data from only 3 CF programs: GTEx, the Human BioMolecular Atlas Project (HuBMAP), and Illuminating the Druggable Genome (IDG); of these three, GTEx is the only program with RNA-seq data from human blood samples. HuBMAP does not have data from blood samples, but Dr. Phil Blood is the director of HuBMAP, so his name matches the search query. IDG also lacks RNA-seq data from blood but does feature a blog post that (separately) mentions both RNA-seq and blood. The other 4 programs that *do* offer RNA-seq data from blood—GMKF, HMP, ExRNA, and LINCS—don't appear at all in the results.

To illustrate why so few relevant data appear in these search results, we used 6 concepts broadly applicable to biomedical data (sample type, general tissue, specific tissue, anatomical part, analysis pipeline, and organism) to manually search the 5 CF program datasets known to have RNA-seq data from human blood. We then documented each program's description of these concepts in their respective data portals. The example search in Table 1 highlights several common types of differences between programs. It is notable that in this search, only 1 value (HMP's ENVO:02000020) uses a Compact Uniform Resource Identifier (CURIE), making it directly linkable to an existing ontology. Most corresponding values are similar to one another but still unique to each DCC.

**Table 1:** An example Google search for Common Fund Data.

A.		ExRNA	GTEx	GMKF	HMP	LINCS
**Sample Type**	**Key**	RNA Isolation Kit	SMNABTCHT	*Analyte Type*	Body Product	Cell line
	**Value**	Serum and Plasma Kit (Quigen)	RNA isolation_PAXgene Blood RNA (Manual)	*RNA*	blood cell	THP1
**Analysis Pipeline**	**Key**	Profiling Assay	SMAFRZE	*Experiment Strategy*	Type	Assay
	**Values**	small RNA-Seq	RNASEQ	*RNA-Seq*RNA-seq	▪ 16s_raw_seq_set▪ 16s_trimmed_seq_set▪ host_transcriptomics_raw_seq_set▪ microb_transcriptomics_raw_seq_set	
**Organism**	**Key**	Species	NA	*NA*	NA	Organism
	**Value**	Human	NA	*NA*	NA	human
						

B.		ExRNA	GTEx	GMKF	HMP	LINCS
**Specific Tissue**	**Key**	Biofluid	SMTSD	*Composition*	Body Site	NA
	**Values**	Plasma	Whole Blood	▪ *Peripheral Whole Blood*▪ *Blood*▪ *Primary Blood Derived Cancer—Bone Marrow*▪ *Blood-Lymphocyte*▪ *Primary Blood Derived Cancer—Peripheral Blood*▪ *Blood Derived Cancer—Bone MarrowPost-treatment*▪ *Blood or Saliva*▪ *Recurrent Blood Derived Cancer -Peripheral Blood*▪ *Blood Derived Cancer—Peripheral Blood, Post-treatment*	blood cell	NA
**General Tissue**	**Key**	Anatomical Location	SMTS	*Anatomical Site (Source Text)*	Supersite	Tissue
	**Values**	Entire cardiovascular system	Blood	▪ *Peripheral Whole Blood*▪ *Peripheral blood*▪ *Blood*▪ *Blood Ficolled Wbc*	blood cell	Blood
**Anatomical Part**	**Key**	NA	SMUBRID	*Tissue Type (Source Text)*	Material	NA
	**Values**	NA	13 756	▪ *Primary Blood Derived Cancer—Bone Marrow*▪ *Primary Blood Derived Cancer—Peripheral Blood*▪ *Blood Derived Cancer—Bone Marrow*▪ *Post-treatment, Recurrent Blood Derived Cancer—Peripheral Blood*▪ *Blood Derived Cancer—Peripheral Blood, Post-treatment*	blood(ENVO:02 000 020)	NA

Table 1 is split in two for ease of reading. For each of the six concepts (A: Sample Type, Analysis Pipeline, Organism; B: General Tissue, Specific Tissue, Anatomical Part) that would be relevant for finding existing datasets for “human blood RNA-Seq,” we list the Key and Value used by each of five Common Fund programs that host this type of data in their search portals. Keys are analogous to column headers in a metadata file, and the values shown are the specific values used at that program that are good matches for this search. NAs indicate that information for that concept is not an available search term at a given portal. GMKF Keys and Values shown as italics denote that while those terms are publicly available, they can only be searched while logged into the GMKF portal, and so do not appear in Google searches.

For each of the 6 concepts we searched, a subtable of Table 1 lists the “key” used by each program to refer to that idea, which is equivalent to the column name in a spreadsheet. “Values” are examples of the data you might find under each key, and here we display values that best fit our “human blood RNA-seq” search. These results show 3 general types of disagreement in term use: differing term values, differing keys in specific categories, and differing assumptions.

Differing term values (e.g., the Analysis Pipeline subtable shows “RNASEQ,” several types of “transcriptomics” datasets, “RNA-Seq,” and “RNA-seq”) hinder effective searching because concepts of interest may not match terms as indexed by search engines. Differing keys (e.g., the same subtable gives “Profiling Assay” vs. “SMAFRZE” vs. “Experiment Strategy” vs. “Type” vs. “Assay”) may not directly impact search, which depends largely on values, but do create problems when combining datasets, since each key must be manually harmonized. Table 1 itself represents one possible harmonization of the given metadata, but other valid choices exist. In the General Tissue subtable, we harmonized HMP's “Supersite” to GTEx's “SMTS” but could equally well have chosen GTEx's “SMTSD,” which we listed instead in the Specific Tissue subtable. With each program using its own (often opaque) terms to describe concepts of interest, researchers trying to make decisions about relevance would need to acquire deep familiarity with each dataset in advance, spending valuable time learning the intricacies of each program's local jargon. According to their documentation, for example, GTEx uses “Whole blood” as a more specific tissue type than “blood” by itself.

Differing assumptions are demonstrated in the Organism subtable. HMP, GTEx, and GMKF only deal with human datasets and do not therefore specify species in their internal metadata, making them more difficult to find for researchers starting in multiorganism contexts. Taken together, the often arbitrary nature of these descriptive differences makes data discovery nearly impossible using a uniform set of search terms.

### A listening tour identified obstacles to interoperation

We conducted in-depth interviews in 2019 with 9 different programs to better understand the obstacles that DCCs face in making datasets more accessible to researchers. We gathered details from each program describing what data they collect, how they model and store that data, and who their target user base is. We used this information to draft essential program requirements and to establish initial working relationships with participating DCCs. During these 2-day, in-person meetings, we typically met with everyone working on the project, including principal investigators, developers, engineers, domain experts, and everyone in between. We published 3 reports describing the results of each individual meeting, synthesizing common themes, and most importantly developed concrete recommendations to NIH to address common needs and encourage DCC participation in the CFDE [14, 16, 25]. To ensure frank discussion, especially regarding difficulties, we anonymized incoming information and gave DCCs advance copies of each report, along with full editorial rights to determine exactly what was (and wasn't) ultimately published.

Despite a wide range of data types, user bases, goals, and project timelines, we found that problems faced by DCCs were both universal and closely related to the Common Fund award structure: all felt they lacked the time, guidance, and funding to meet all the mission needs of their consortia. The Common Fund vision was to act as a risk-tolerant startup fund for cross-cutting, ambitious projects. Failing programs would be quickly disbanded, while successful ones would be folded into other more stable NIH funding sectors. DCCs were therefore by design strictly limited to 10 years of CF funding, disbursed in yearly, evenly sized amounts as part of mutable awards that could be edited to shift consortia in new directions as needed.

In principle, this should have allowed researchers to work on bleeding-edge scientific methods not typically funded by NIH. In practice, however, it led to a series of underfunded data silos and an unknown amount of lost data. Due to the novelty of each project, the Common Fund could not provide detailed guidance for how to operate each DCC or develop operating standards that were generally relevant. As DCCs raced to ramp up and show progress in their early years, they had neither time nor staff for extramural interactions, a situation only exacerbated by the lack of any CF-wide venues for cross-program interactions and awards all beginning at different times, subject to different goals. Across our 9 visits, only 1 DCC could even *name* another Common Fund DCC. Each DCC thus chose (and often created) completely different and mutually incompatible standards, workflows, and data descriptors (as demonstrated in Table 1), even when similar work was being done in other CF programs.

Because funding was not only time limited but also metered to the same amount each year, DCCs found themselves flooded with early funding and then wildly underfunded by budgets that did not adapt to evolving priorities. Most were not able to hire and begin work fast enough, thereby losing portions of funding in their first and second years. As each DCC matured and took on more data, staff, and responsibilities, the metered yearly funding increments then proved too small, leaving important work undone. The work left undone, in nearly every case, was future planning to ensure data were preserved and made available to the research community after the end of each program's funded life span. As a result, at the 10-year mark, some DCCs kept their data available by lumping them into other funded projects, and other DCCs simply stopped, as they had not the time or staff to establish ongoing curation and data provisioning.

All of the DCCs we interviewed were interested in increasing interoperability, but all lacked the resources to do the deep cross-program collaboration required to build the shared systems that could realize this goal. Our recommendations therefore concentrated on issues of time, money, and collaboration, and the Common Fund Data Ecosystem Coordinating Center (CFDE-CC) was thus established as a kind of DCC for DCCs. The CFDE-CC assumed all collaboration overhead: introductions, cross-program meetings, physical logistics, and so on, so that DCC participants could simply show up to meetings and discuss strategies for interoperation. We also worked with the Common Fund to establish supplemental funding streams for participating DCCs to cover the costs of developing collaborations and data management plans and worked one-on-one with DCCs nearing the end of their funding to move their data into CFDE-funded cloud storage so they would not be lost.

Our listening tour also produced first steps toward improving CF-wide searching and interoperability of program resources. From our discussions, we were able to identify data elements programs had in common (see Table 1 for examples) that could become the basis for the C2M2, an overarching metadata model to describe datasets managed by all current DCCs. Over the following 2 years, we built a collaborative consortium of invested DCCs, elaborated the C2M2 through a consensus-driven process, instantiated the C2M2 in a rich relational database, ingested C2M2-harmonized project metadata from 11 participating DCCs, and built a unified web-based portal interface to this new cross-program project catalog.

### Entities and relationships are the key structural features of the C2M2

The Crosscut Metadata Model comprises a group of metadata concepts (entities) that describe key components of biomedical research results. Entities are linked one to another via well-defined relationships. From our listening tour, we identified a small set of critical concepts for describing research results that were common across multiple DCCs. We modeled our first set of C2M2 entities around these concepts (although it is important to note that this set was neither universal nor exhaustive). Three of these initial entities represent tangible experimental resources: *files* (digital bytestreams encoding experimental data), *biosamples* (living material collected and processed via experimental protocols), and *subjects* (organisms studied experimentally, both directly observed and as biosample donors). Two additional entities serve to aggregate these tangible resources into meaningful groups: *project* (very broadly representing funded research studies governing the experiments being described) and *collection* (a generalization of “dataset” that can include representations of biosamples and subjects in addition to the files that typically exclusively comprise a dataset). We refer to C2M2 project and collection entities as *containers*: named sets or groups *containing* particular C2M2 files, biosamples, and/or subjects. Containers were designed not only to allow DCCs to explicitly and flexibly group related entity records into named sets but also to associate these groups with well-defined scientific concepts (described in the next section).

Entities are physically represented in C2M2 as *tables*: rectangular matrices consisting of ordered rows (*records*) and columns (*fields*). Each record is a small list of named pieces of metadata (fields), which, taken together, comprise that record. Each record in a given table describes one particular instance of whichever entity that table represents: 1 row/record in the “file” table, for example, represents 1 individual file, with the entire “file” table thus representing the set of all individual files being described. Each *field* in each record comes with a predefined meaning that helps to describe some key aspect of that record (for example, file records have fields describing file size, file format, and file name, among others).

**Figure 1:**
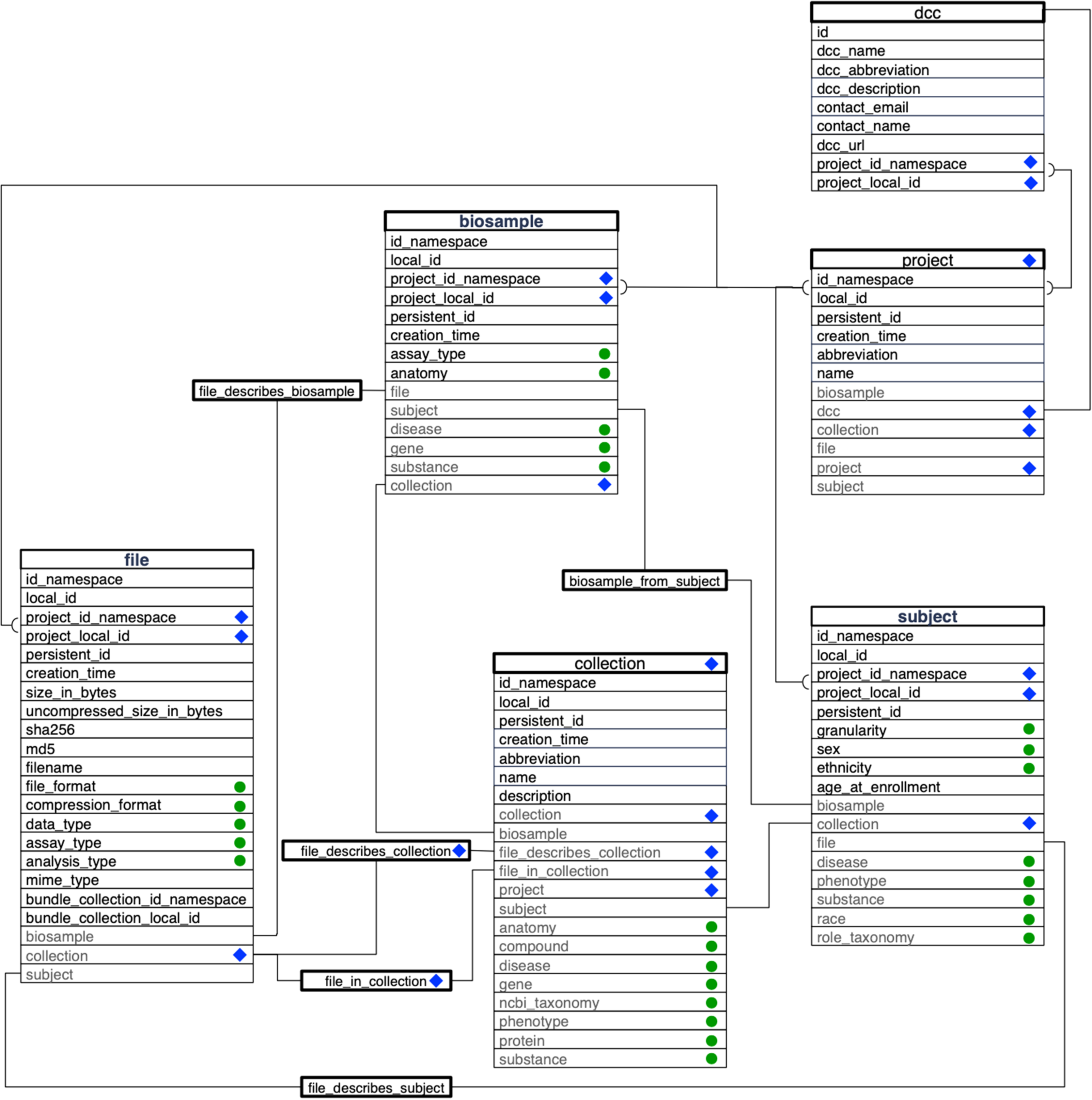
A simplified entity relationship diagram for the C2M2 where most association and controlled vocabulary tables are collapsed into the main entity tables. Each entity is shown as a large table; fields outlined in black are columns of the tables in which they appear; fields labeled with blue diamonds abbreviate connections to container entities and their related associations. Fields marked with green circles indicate controlled vocabulary terms, and fields outlined in gray indicate association-table connections to entities named therein. Gray-bordered fields with green circles indicate connections between bare CV terms (included in the entity tables) and master controlled vocabulary term tables, which track global term usage (term tables are described in detail in the Results section). Lines are drawn connecting fields that participate in interentity relationships. Boxes on these paths name association tables that instantiate these connections but do not explicitly list those tables’ fields.

Relationships *between* entities are represented as *association tables*, whereby individual metadata records describing different entity types are linked to one another according to broad relationship definitions, like “file X describes biosample Y” or “biosample K104 came from subject S7786.” Fig. 1 shows a simplified *entity-relationship (ER) diagram* focused on the tables for the 5 entities mentioned above (file, biosample, subject, project, and collection), along with a “dcc” entity recording basic provenance information for each CF program DCC that submits C2M2 data to the central database. See Supplementary Fig. S1 for the full C2M2 ER diagram describing all 48 tables (both entities and associations).

Common Fund DCCs prepare C2M2 *instances or submissions*—chunks of metadata describing their experimental resources—in the form of groups of table files corresponding to the components detailed in the ER diagrams referenced above; these files are validated by CFDE software and then *ingested* into the central C2M2 database for publication via the CFDE web portal. Valid C2M2 submissions must provide minimal information wherein each C2M2 entity record that represents a tangible experimental resource (like a file or a biosample) is linked to exactly 1 C2M2 *project* record, which in turn describes the research effort under which the tangible resource (file, biosample) was created or observed. Essential benefits and functions (findability, reusability/citation, interoperability, searching, etc.) depend on this information, so that as C2M2 resource information is discovered and used by users, it is properly associated with its originating research context. DCCs can optionally aggregate C2M2 records into *collections* (generalized named datasets): because of their very general structure (“a thing that contains other things”), decisions that precisely define the scope and complexity of these collections can be left to each submitting DCC. Collections can also optionally be assigned persistent IDs (like DOIs) for stable citation, reference, and retrieval.

A C2M2 record need not (and generally will not) have values for every possible metadata field in order to be usable, especially as the C2M2 broadens to accept new data types and variants: most C2M2 field values are optional. Nearly every field, and in most cases entire tables, can be left blank, allowing each DCC to customize their C2M2 submission to whatever level of richness or focus best fits their capacity and presentation goals, while meeting basic universal criteria designed to permit interoperation and discovery that are kept conservatively minimal by design.

### The C2M2 integrates standardized vocabularies

A key component of cross-DCC metadata harmonization within the CFDE is support for the detailed description of C2M2 metadata using terms from standard scientific ontologies. C2M2 currently provides a variety of (optional) fields and tables through which controlled (standardized, curated) scientific vocabulary terms can be attached to C2M2 collections and entities. Currently supported concept vocabularies include the Disease Ontology [26]; the Ontology for Biomedical Investigations [27]; the Uber-anatomy Ontology (UBERON) [28]; the NCBI Taxonomy [29]; EDAM [30], an ontology for bioinformatics concepts including data types and formats; gene terms from Ensembl [31], a database for researchers studying genomics in humans and other vertebrates and model organisms; PubChem [32] the world's largest curated cheminformatics database; GlyTouCan [33], a glycan structure repository; the Human Phenotype Ontology [34], a collection of terms describing various external conformations of human anatomy; and UniProtKB [35], the world's largest collection of protein metadata, combining both expert-curated and auto-annotated information. An exhaustive list appears in Table 2.

**Table 2:** Controlled vocabularies currently supported in C2M2

CV field or association	Ontology	Description
file.analysis_type	OBI	The type of analytic procedure that produced a file
file.assay_type	OBI	The type of experiment that produced a file
file.file_format	EDAM	The digital format or encoding of a file (e.g., "FASTQ")
file.compression_format	EDAM	The compression format of a file (e.g., "bzip2", “gzip”)
file.data_type	EDAM	The type of information contained in a file (e.g., "sequence data")
biosample.assay_type	OBI	The type of experiment that produced a biosample
subject_phenotype	Human Phenotype Ontology	Link subjects to phenotypic observations
biosample.anatomy, collection_anatomy	UBERON	The physiological source location in or on a subject from which a biosample was derived, or an anatomical part relevant to a particular collection
biosample_disease, subject_disease, collection_disease	Disease Ontology	Link biosamples, subjects, and collections to observations about diseases
biosample_gene, collection_gene	Ensembl	Link biosamples and collections to individually relevant genes (e.g., knockdown targets)
biosample_substance, subject_substance, collection_compound, collection_substance	PubChem, GlyTouCan	Link biosamples, subjects, and collections to drugs, reagents, other small molecules
ncbi_taxonomy.id, subject_role_taxonomy, collection_taxonomy	NCBI Taxonomy	Link subjects or collections to taxonomic names
collection_protein	UniProt KnowledgeBase	Link collections to individually relevant proteins

Entity term fields are listed as C2M2_entity_table.field_name; term association tables (one-to-many relationships between entities and vocabulary terms) are listed by table name. We give the source ontology for each vocabulary, along with a general description of its annotation role within C2M2.

If sufficiently specific terms cannot be expressed using C2M2-supported ontologies, we encourage DCC data managers to contact the CFDE Ontology Working Group (OWG) with proposed additions to the supported vocabulary sets. The OWG has established direct update channels with the curation authorities for each supported ontology, and this working group routinely expedites the addition of missing terms on behalf of CF DCCs. The CFDE portal supports discovery based both on official vocabularies and also on any new provisional terms awaiting approval, so DCCs can add usable preferred terms to their C2M2 submissions immediately, rather than using terms with poor fits or leaving fields blank altogether. We also note that the same infrastructure that facilitates this enhanced synchronization with C2M2-supported vocabularies, allowing the incorporation of provisional terms into the C2M2, can also be used to maintain obsolete terms in cases where the original dataset cannot be updated but subsequent curation of the relevant vocabulary has removed them.

### The C2M2 supports a flexible system of internal and global identifiers

The C2M2 is designed to be a framework for sharing information with the global research community about data deriving from experimental resources. Experimental metadata are created at different times by different DCCs working independently, so any system trying to federate such information must establish a standard way for DCCs to generate stable identifiers (IDs) without requiring DCCs to coordinate ID usage directly with each other. At the same time, any integrated system must guarantee unambiguous, internally consistent IDs, so a minimally effective ID scheme must allow DCCs to create IDs for their C2M2 metadata objects that do not (and will never) clash with C2M2 IDs created by other DCCs (some of which may have since ceased to exist).

The C2M2 provides 2 types of entity identifiers: a mandatory *C2M2 ID* and an optional *persistent ID*. C2M2 records for files, biosamples, projects, subjects, and collections must each be labeled with a C2M2 ID. A *C2M2 ID* has 2 parts: a prefix (id_namespace) representing the source DCC and a suffix (local_id) representing the specific entity record (individual file, project, etc.) that the C2M2 ID identifies. The 2 parts of each C2M2 ID, concatenated, serve as a unique ID for each record that is guaranteed to be unambiguous across the entire CDFE ecosystem: because each ID is prefixed with a unique portion identifying the contributing DCC, this scheme allows DCCs to directly import their preferred (intramural) ID scheme directly into the local_id component, generally without modification. The optional *persistent ID*—stored separately from C2M2 IDs—is a URI that encodes actionable information that users or automated software can follow to get more information about the resource named by the ID, potentially including access or download details. The CFDE system accepts a wide variety of persistent ID types, including minids [36], Data Repository Service (DRS) IDs, and digital object identifiers (DOIs); see the Identifiers Supplement for details.

### Independent “data packages” are submitted to the CFDE

The C2M2 is designed to integrate asynchronous submissions from multiple programs, operating independently. Each submission comes as a "data package,” a collection of data tables encoded as tab-separated value files (TSVs). Each DCC collects metadata describing their program's experimental resources into a single data package that it then submits to the CFDE. DCCs can explore submitted data packages in advance of publication using protected areas of the CFDE portal: the system will accept multiple submissions between public data releases, with only the most recent (approved) version of each DCC's data package published as part of each periodic portal release.

A C2M2 data package consists of 48 TSV files (as of 4 April 2022) populated with interrelated metadata about DCC data assets. Precise formatting requirements for data packages are specified by a JSON Schema document that describes the entire C2M2. The C2M2 JSON Schema document is itself structured according to the “Data Package” meta-specification published by the Frictionless Data group [37]: implementing this structural standard enables DCCs and the CFDE to leverage an existing ecosystem of validation tools. The C2M2 specification defines foreign-key relationships between metadata fields (TSV columns), rules governing missing data, details governing required content types and formats for individual fields, and other constraints. These architectural rules ensure the internal structural integrity of each individual C2M2 submission. Most important, they also serve to guarantee and maintain a unified, harmonized C2M2 database underpinning the CFDE portal, automatically enabling compatibility and interoperability across multiple federated submissions received from different DCCs at different times.

Data packages can be created with different levels of complexity: many columns and several entire tables can be left empty and still produce a valid package for submission. Only 3 metadata records (3 rows, across 3 C2M2 tables) are strictly required, so most tables can be left empty in a minimally compliant submission. The 3 required records are:

1. a short contact record referencing the submitting DCC,
2. a single project record representing the submitting DCC (for resource attribution), and
3. at least 1 ID namespace, registered in advance with the CFDE, that protects record IDs from conflicts with other DCCs’ metadata (see previous section).

The simplest *usable* submission configuration will also contain at least 1 data table listing an inventory of tangible experimental resources (e.g., files). A more complex submission might inventory additional entities and might also encode basic relationships between those entities. For example, a submission might record which biosamples came from which subjects or which files contain data pertaining to particular biosamples. DCCs can further opt to group their C2M2 metadata into collections, describing datasets *per se*, or (more powerfully) associating resources with specific scientific concepts (e.g., “these resources all relate to aspirin” or “this set is about human gene FMA1”).

### Preparing a project submission for ingest into the catalog

Each TSV in a C2M2 submission is a plain-text file representing a tabular data matrix, with the first (*header*) line listing tab-separated column names and any subsequent lines representing table rows (records) with tab-separated column values (fields). TSVs must conform to all formatting and relational constraints in the C2M2 JSON Schema document [38]. Any blank table is submitted as a TSV containing just the header line—requiring inclusion of these stub TSVs helps differentiate between intentional and accidental data omission.

For each controlled vocabulary supported by C2M2, a *term table* must be included (see Fig. 1 [fields with green dots] and Supplementary Fig. 1 [green tables]). Each such table will contain 1 record for each controlled vocabulary term used anywhere in the C2M2 submission, along with basic descriptive information for each term that helps match user searches and populate result display pages in the portal. Once the entity and association tables are prepared by the submitting DCC, the CFDE-provided *submission preparation tool* [39] scans the prepared tables for controlled vocabulary terms, validates all found terms against CFDE-maintained ontology reference files, and automatically builds all necessary term tables (as TSVs), importing needed descriptive information directly from the ontology reference files. These automatically generated term tables are then bundled along with the rest of the C2M2 submission.

### The CFDE provides robust data ingest and validation for data packages

**Figure 2:**
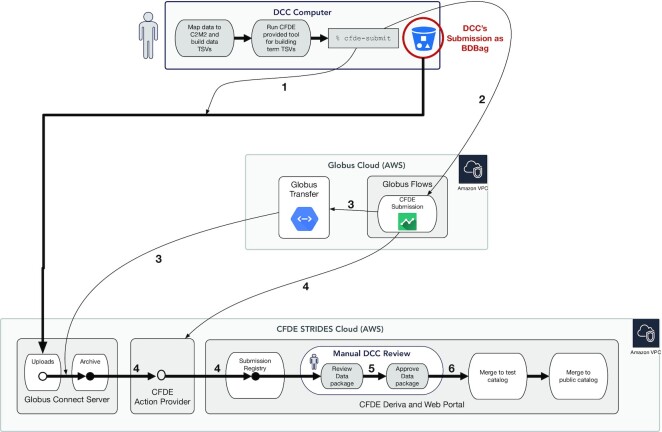
CFDE submission process. A DCC initiates the submission process by creating a new set of TSVs that meet the C2M2 requirements, running a CFDE tool to build term tables, and submitting that entire datapackage. The cfde-submit CLI then performs a lightweight validation of the submission data, starts the data upload to CFDE's servers (step 1), and then initiates processing in the cloud (step 2). The system that manages the cloud processing is called Globus Flows. Globus Flows is Globus software-as-a-service (SaaS) running in the AWS cloud. CFDE's submission process is one of many “flows” that the flows service manages, and the final action of cfde-submit is to start a run of the CFDE submission flow. The CFDE submission flow moves the submitted data to a permanent location (step 3), sets access permissions (not shown), and executes code on a CFDE server (step 4) that ingests the submitted data into the CFDE portal's database service, Deriva. While processing is happening in the cloud (steps 2–3), status can be checked using cfde-submit, but it does not appear in the CFDE portal until step 4. At this point, the DCC uses the CFDE portal to review and approve (or reject) the datapackage (step 5). Deriva then merges the new datapackage into a test catalog before finally publishing it to the public catalog (step 6), making it searchable by anyone at the CFDE portal.

The CFDE-CC provides a full-service submission system for incoming C2M2 data packages. DCCs send submission files to this system using the *cfde-submit tool*, a lightweight command-line Python package [40] that performs authenticated upload to the CFDE portal database via Globus Flows [41] (Fig. 2). The tool scans a directory of C2M2 TSVs, validates their contents against the C2M2 JSON Schema, and builds a BDBag file [42] containing the TSVs, a copy of the schema, and some minimal provenance metadata. The tool then securely uploads the BDBag to a dedicated DCC-specific Globus [43] endpoint provided by the CFDE-CC during onboarding; only explicitly authorized DCC users can submit to their DCC's endpoint.

Once a data package is uploaded, a DERIVA [44] database instance automatically begins ingesting it, performing further validation using a custom validation script. Users are notified by email when the ingest process has completed and are provided with a link to preview the data in a secure section of the CFDE portal (or to access a description of any ingest errors). Following review, edits, and explicit approval by the submitting DCC, each submission is set to be merged into the central CFDE database, to be made viewable and searchable in the CFDE portal (as part of ongoing quarterly releases). Figure 2 gives an overview of the submission workflow.

**Figure 3:**
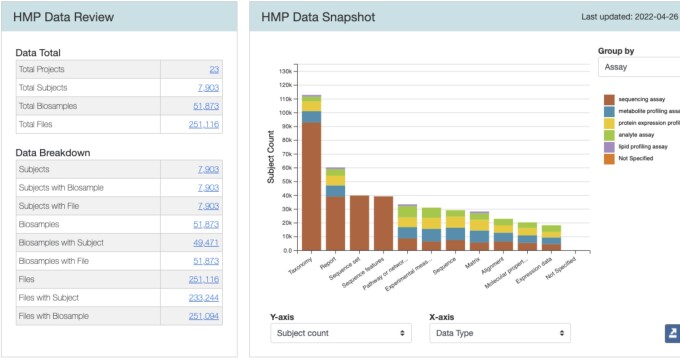
Summary page of a submitted data package with interactive chart and summary statistics.

To make changes after submission and before public release—for error correction, or to experiment with different ways of modeling and presenting their data—each DCC can submit multiple successive data packages, using a secure section of the CFDE portal to view each submission in various ways. DCC staff can (privately) interact with submitted data exactly as they will be released via the public CFDE web portal; they can also access an interactive high-level overview of each submission along with various summary statistics (Fig. 3). Graphics describing internal data package connectivity (bottom of Fig. 3) summarize various aspects of user findability for submitted data: if 100% of a submission's file records are annotated with “data type” metadata, for example, then portal users will be able to find all the files in the submission when searching by data type.

All CFDE metadata releases include exactly 1 submission from each participating DCC: on every (quarterly) release date, the newest approved submission from each DCC becomes findable, browsable, and searchable in the public CFDE portal. If a DCC does not submit and approve an updated C2M2 submission between releases, their most recent public submission remains accessible without modification; if a new submission is approved, at the next release, it will completely replace that DCC's previously available data. Incremental (partial) data updates are not supported: they are generally error prone and create increasing difficulty over time for DCC curators trying to maintain guarantees of stable data provenance and reusability.

### Users can query the combined C2M2 catalog using the CFDE portal

DCC-approved data packages are merged quarterly into a publicly accessible CFDE portal database (“catalog”). This catalog is a customized instance of DERIVA [44], an asset management platform that provides both a model-driven web interface and a command line interface (deriva-py), with a relational database back-end schematically designed to store C2M2 metadata along with associated supporting information required by the portal interface.

The CFDE web portal supports 3 basic types of search:

1. Search to find records of specific types, with results *interactively faceted by user-selectable lists* of all associated scientific and technical vocabulary terms; for example:
  • Show me *files* (select a specific record type)
    ○ …now narrow those down to only *FASTQ* files (filter on file format)
    ○ …then winnow those results to include only FASTQ files associated with *transcriptomics assays* (filter by assay type)
    ○ …then further filter those results to include only data associated with *kidney* (filter by anatomical part)

In each step above, the user refines their search by selecting values from a *facet* or *dimension* (“file,” “file format,” “assay type,” “anatomy”) which is presented to them as an interactive list as they browse the CFDE portal seeking information of interest.

2. Search to find records of specific types via *free-text search* on names, descriptions or synonyms of scientific terms, projects or collections associated with those records; for example:
  • Show me *biosamples* (again, select a specific record type)
    ○ …now find me biosample records with the word *aspirin* appearing anywhere in their associated metadata

In this case, the user selects a record type (biosample), then types “aspirin” as a search keyword; the portal will then find all biosample records that have been associated with that keyword in any of several possible ways; for example:

• the biosample is in a *collection* annotated with PubChem term #2244 (aspirin)
• the biosample is in a *project* whose name includes the word “aspirin” (e.g., “Aspirin Metabolomics in Colorectal Cancer Prevention”)
• the biosample came from a *subject* who had been administered aspirin as part of a clinical study

3. Search to find records associated with a *single controlled vocabulary term*; for example:
  • Browse & search the entire list of “anatomy” terms to identify all such terms with the word “blood” appearing anywhere in their description
  • …then select “umbilical vein” as a term of particular interest
  • …then view all biosamples, collections, etc., associated with “umbilical vein”

These search modes are motivated by both practical and scientific considerations. From a practical standpoint, types 1 and 2 guide users to begin with a small set of general entity types (file, biosample, collection, subject, or project) and then make successive choices to refine those searches, producing manageable sets of relevant records. Due to the wide range of data types hosted by Common Fund DCCs, these are the 5 record types that all DCCs generally share: by focusing these search modes on these concepts, we expect that users will be able to most easily find data from all participating programs. As more programs are added, new entities will be created with new associations formed between them, and new search types can be added to leverage these new data.

Scientifically, we heard both in our listening tour and in ad hoc discussions with researchers that even establishing the *existence* of data meeting desired scientific criteria is a big challenge. The only current options are laborious literature searches prone to false positives or trying to individually search as many data repositories as possible—which we have seen (Table 1) is difficult at best and often impossible to do in any comprehensive way. Bioinformaticians are willing to go back to original data sources to learn more about datasets of potential interest—indeed, they typically must do so, in order to properly use the data for further analysis—but they are naturally eager to streamline the frustrating steps involved in simply *identifying* data of potential interest in the first place.

The first 2 search types support cases where users are interested in finding instances of specific asset types (files, biosamples, subjects, or, more broadly, whole projects or collections). Using these modes, a researcher chooses an entity of interest and filters from there with a simple faceted search, a faceted search using Boolean operators (“include X,” “exclude Y,” etc.), or free-text search, to home in on specific resources of potential interest to them. Such searches are especially useful for researchers looking for data similar to that produced by their own experiments.

Users interested in finding data related to a specific assay type, tissue type, disease, gene, chemical compound, or other well-defined scientific concept (represented in the CFDE portal as controlled vocabulary terms) can use the third search type to filter the CFDE catalog to show all resources associated with selected concepts of interest. This mode allows researchers to quickly assess whether specific data (e.g., mass spectrometry data or psoriasis data) *exist* in any Common Fund dataset, without needing to know in advance what specific type of asset(s) the data are associated with.

Researchers can search the CFDE portal anonymously without registration or can register to access a dashboard page where they can view interactive summary plots, save and replicate customized searches, and build personalized collections of “favorite” items. Registered users can log into the CFDE portal using a number of identity providers, including eRA Commons.

### Content searchable at the CFDE portal continues to expand

**Figure 4:**
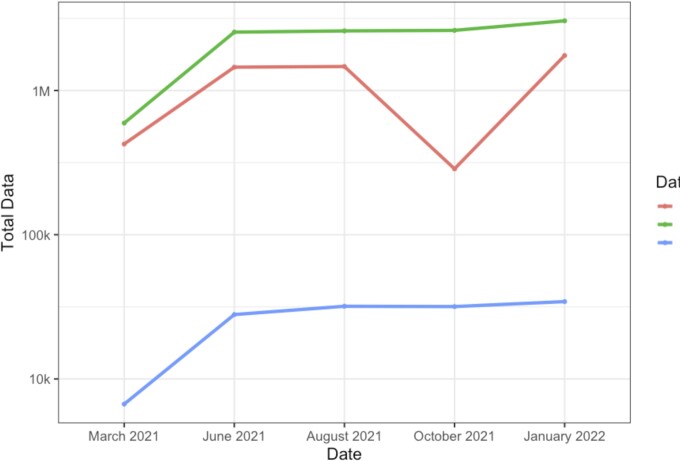
Core data available for search at the CFDE portal over time. The sharp decrease in biosamples in October 2021 is due to replicate cell line data being more appropriately modeled as from a single biosample. Note that the y-axis is exponential, and therefore the increases are quite large: the January 2022 release, for example, contains nearly half a million (430,405) more files than the October 2021 release.

Following a series of internal prototypes, our first limited portal release with live data, on 30 March 2021, included submissions from 7 Common Fund programs. This first release allowed our beta-test researchers to search across a combined 594,507 files, 425,341 biosamples, and 6,689 subjects (Fig. 4). As of our first public release in February 2022, the CFDE makes 3,041,978 files, 1,749,145 biosamples, and 34,375 subjects from 11 programs searchable in a single harmonized interface.

The C2M2 is a living standard and is constantly being expanded to allow new datatypes, new associations, and better ways of describing the underlying data. Over time, DCCs also get better at using the C2M2 to describe their data, a phenomenon clearly visible in the changing number of biosamples reported over time in Fig. 4. In early versions of the portal, DCCs treated cell line replicates as unique biosamples; as of October, replicate names were merged so that the search interface will return all uses of a given named cell line. While not public, improvement is also evident from browsing the review pages for data submissions from oldest to newest. For every participating DCC, we see increasing interconnectivity within submissions in addition to increases in the overall amount of metadata submitted.

### The C2M2 and the portal are evolving over time to better support search

In the first iteration of the CFDE portal, metadata search was a direct extension of the C2M2 model. Once DCCs began submitting data, it quickly became evident that we needed to provide an extra layer of abstraction between the C2M2 model and users to support intuitive search. The reason was that the terms that DCCs used to describe data often did not correspond to the terms employed by users to search for data.

An example of controlled vocabulary anatomy terms can illustrate this difficulty. C2M2 uses UBERON for these terms; each DCC maps their local anatomy specifications to UBERON for inclusion in their data package. We initially expected this mapping process to harmonize anatomy terms across DCCs for easy unified search, but we immediately discovered that there was no more overlap in term use when the DCCs all used the same vocabulary than when they each used their own idiosyncratic vocabulary. UBERON has, at present, 21,911 unique anatomy terms, many describing subtle shades of difference in anatomical structure; when deciding which UBERON terms best matched their local terminology, DCCs often chose terms with different levels of specificity. Similar results were observed across all the C2M2 controlled vocabularies: a unified vocabulary does not sufficiently constrain usage to automatically produce harmonized results.

We instituted 2 new practices to compensate, one social and one technological. The social solution was to create a working group to formulate best practices regarding use of C2M2 ontologies and controlled vocabularies, wherein DCC members can discuss and agree on community guidelines for term selection that simultaneously produce good fits to their data and maximal meaning for portal users. Even with the guidance of best practices, though, differences in usage are inevitable, so we also developed a layer of abstraction in the portal that lets users search on higher-level, more general scientific terms, under which more specific terms can be grouped through the use of “slim maps” (or “slims”) [45]. For the UBERON anatomy, these “slimmed” search terms are mostly system-level names such as nervous system (UBERON:0001016) and connective tissue (UBERON:0002384). These maps support maximum system-wide flexibility, allowing DCCs to use specific terms to precisely describe their data, while also letting users search on general terms without losing access to relevant results.

In the CFDE portal, users can now choose to search on all anatomy terms, only slimmed terms, or both. Similar slim search capabilities have also been implemented for other C2M2 controlled vocabularies.

### The C2M2 and the portal continue to evolve to improve user experience

A known challenge in the design of user interfaces to complex systems such as Common Fund data repositories is to ensure that users intuitively know how such systems should be used. To help maximize findability in the CFDE portal, we conducted 2 rounds of usability testing for the portal interface since it launched. Hour-long, in-depth interviews were conducted by a professional user experience team [46] to measure both how users currently interact with the portal and also how they would like to interact with it. This process exposed a number of assumptions and preferences that in turn inspired interface refinements to enhance user experience. Key elements sought by users included (i) highly specific terminology or controlled vocabulary; (ii) summaries of types and extent of data available within the portal; (iii) the ability to easily find and complete key tasks; (iv) consistent, contextual navigational elements; (v) easily comprehensible data visualizations; (vi) the ability to compare tabular data against data visualizations; and (vii) dates of data submission. All of these features, along with many others suggested during these interviews, have been implemented or enhanced and will be subject to further refinement driven by the next testing cohort.

## Discussion

The CFDE portal is a central search solution for locating and exploring Common Fund datasets. While researchers searching for data experience a simple, intuitive user interface, this interface is powered by a sophisticated underlying architecture, built in layers on top of a database implementing the harmonizing C2M2. The CFDE-CC manages and maintains this infrastructure, whose usefulness ultimately depends on a growing federation of Common Fund program DCCs that regularly submit detailed research metadata, geared to connect the global research community with Common Fund data. This critical dependence has driven fundamental design decisions for the CFDE, including incremental adoption of the C2M2, specification of standards and best practices, our approach to evolving and extending the C2M2, and the overall structure of our social and technical federation.

### Data submission is supported and subsidized by the CFDE ecosystem

On the technical side, participating DCCs create their submissions by mapping their internal data models to the C2M2. Depending on the complexity of their data, this mapping can be a difficult task: this was a concern heard repeatedly in our listening tour, and addressing DCC participation costs was a core recommendation of our final tour report [16]. As a result, the CFDE is designed to provide full support for data submitters. Critically in this respect, all DCCs receive supplemental NIH grants through the CFDE project to offset the costs of building their data packages and participating in working groups [47]. To support DCCs in creating their data submissions, we publish and maintain detailed technical documentation [48], a novice-friendly wiki [49], a bug and request tracker [50], and a full-service helpdesk. We also provide tooling for every automatable step of the submission process.

Harmonizing scientific descriptions of research data with the C2M2 requires deep knowledge of DCC source data and is the most human-intensive step. Once DCC staff have mapped local descriptions to the C2M2 and enumerated their data, we provide tooling for automatically building term tables [39], packaging and submitting data [40], and reviewing submitted data before publication [23].

The CFDE as a whole also offers substantial social support, decentralized with minimal reliance on the CFDE-CC. Our position at the CFDE-CC is that if we do our jobs correctly, those jobs will cease to exist. Our goal is to build a self-sustaining community that actively works toward interoperability as each new DCC begins its funding life cycle, so that integration challenges documented on our listening tour become relics of the past. As such, the CFDE-CC provides both technical infrastructure and administrative logistics (calendering, meeting space, C2M2, portal, etc.) to support DCCs coming together to make decisions and harmonize data. CFDE working groups guiding critical aspects of the ecosystem are proposed, chaired, run, attended, and dissolved by the DCCs themselves. These groups have allowed the community to flexibly focus on short-term goals, as well as establishing forward-leaning conversations about emerging challenges and new directions, driven entirely by the DCCs.

### The C2M2 and the CFDE portal improve FAIRness

Bioinformaticians have at their disposal a wide variety of reliable tools for taking in data and producing results, but this is only one small part of data analysis. Finding, understanding, cleaning, and harmonizing data—prior to analysis—consume a huge amount of time and expertise (and hence expense), and these were some of the core problems that FAIR (findability, accessibility, interoperability, and reusability) was designed to address. The C2M2 and the CFDE portal mitigate these challenges both by improving the FAIRness of Common Fund data and also by centralizing the burden of harmonization. When a DCC builds a C2M2 representation of their data, they are simultaneously making that data more uniformly findable and giving their expert opinion on how their data relate to community-standard scientific concepts, via controlled vocabularies. DCCs can include data locations and access requirements in their C2M2 metadata, increasing data accessibility. When a DCC maps its research metadata to the C2M2, that metadata are *de facto* harmonized with every other participating DCC, greatly enhancing interoperability. Global researchers using the CFDE portal can streamline previously costly and laborious tasks of trying to identify Common Fund data of interest and fairly comparing different datasets to one another and instead use the expertly mapped metadata in the C2M2, making Common Fund data more reusable. Taken together, easily found data harmonized by DCCs should result in a greater number of studies that are higher quality, more comparable, and more easily reproducible, as everyone who reuses the data will use expertly curated and fully interoperable data descriptions.

### The C2M2 is designed to support incremental adoption

C2M2 is designed to support incrementally expanding use by participating Common Fund programs. Its modular, extensible structure facilitates the graded introduction of metadata from CF programs into the CFDE system, through submission of data packages with gradually increasing size, complexity, and detail. As program DCCs contribute more detailed metadata descriptions of their data, global research users of the CFDE portal can conduct more sophisticated searches to better locate resources of relevant interest. The most basic C2M2 submissions can consist of simple asset inventories. Over time, basic inventories can—through a succession of modest improvements—gradually become well-decorated networks of resources and interrelationships between resources. Most important, these improvements can be done by incrementally *adding* detail without having to refactor existing metadata.

Our philosophy of incremental adoption is also intended to address the needs of different DCCs that inevitably operate at widely different scales of data complexity or funding, as well as to simultaneously accommodate different life-cycle phases, research scope, and so on. DCCs with advanced, operationalized metadata modeling systems of their own should not encounter arbitrary barriers to C2M2 support for more extensive modeling of their metadata if they want it; newer or smaller DCCs, by contrast, may not have enough readily available information to feasibly describe their experimental resources beyond giving basic asset lists and project attributions. By committing to maintaining C2M2 as a system of modular extensions, we can swiftly incorporate more complex DCC metadata as needed while also offering well-structured and harmonized options for simpler metadata submissions. One benefit of this approach has been to minimize barriers to rapid entry into the C2M2 ecosystem and its downstream applications, regardless of where each DCC is with respect to curation complexity and funding.

The interface for DCCs to review submitted C2M2 metadata in the portal complements this process of incremental improvement without bias regarding what specific information is included at any particular stage. DCCs manage very different types of data, so any individual submitter will find some tables are impossible to fill, some connections impossible to make, and some entity types irrelevant to their own resources. As an aggregator of aggregators, the CFDE cannot prescribe in advance what details any given DCC “should” provide: instead, we trust our partners’ expert familiarity with their own data to drive decisions about what to include and act as a support system to help make that data coherently available via the CFDE portal. The DCC-accessible data review section of the portal thus aims not to score or rate individual submission but rather to offer information that can be used to understand and track their self-driven data updates. The DCC in Fig. 3, for example, has 0% connectivity between biosample records and chemical substances, but that is because this DCC has no pharmacological intervention studies and so has no substances to list. By presenting information for review without judgment, submitters can both check that data were uploaded correctly and also assess submissions with respect to their own particular data configurations. All submitted data packages are retained indefinitely in the portal, so submitters can compare metrics for previous submissions with new ones to analyze the evolution of their data on their own terms.

The ability to submit C2M2 metadata in managed stages of sophistication serves 4 important purposes. First, it flattens the learning curve for onboarding of DCC data managers by making it possible to quickly create useful submissions with little effort. Second, it lets DCC data managers test how ecosystem-wide realities (overlap in scientific term usage across the entire CFDE, for example) impact their C2M2 metadata before investing more heavily in creating more complex submissions. Third, it allows DCCs to provide feedback to CFDE on how to modify C2M2 over time to better suit individual curation needs. Fourth, incremental extension makes data submissions both forward and backward compatible: changes to the C2M2 add optional new tables, or include new scientific vocabularies, but avoid modifying the existing model structure, so valid submissions generally remain compatible with future C2M2 versions, even after DCCs reach the end of their active funding cycles.

### The C2M2 interfaces seamlessly with existing standards

The world has no shortage of standards, and we have specifically designed our model to leverage mature scientific and technical standards wherever possible. Ultimately, a successful metadata model is one that fulfills community needs. In keeping with this philosophy, our initial version of the C2M2 was an evolved version of the DATS model [51], where each data contributor used somewhat different encodings to describe their data using an ultra-flexible system [52]. During in-depth interviews conducted during our listening tour, however, we were able both to determine the specific needs of each DCC for modeling their data and also to learn what metadata were most important to their users. Synthesizing this information indicated a strong general desire for harmonization, so we completely reimplemented the C2M2 to rely heavily on controlled scientific vocabularies (Table 2) and to require a common set of metadata tables. This eliminated the anything-goes flexibility of the previous model, but the current approach has proven more than nimble enough to meet the needs of the CFDE community, while also supporting the types of faceted search that biomedical researchers prefer.

With respect to C2M2, all 4 components of FAIRness are enhanced by the integration of established standards directly into the model framework. Findability is created through the use of common terms to describe common scientific concepts. Accessibility benefits from integration of technical standards that allow users uniform access to heterogeneous data sources without having to cope with multiple bespoke interfaces. Interoperability is defined by how well data flows interact with other information systems, which is obviously only improved by the integration of common descriptive standards. Reusability depends both on persistence of data over time (encouraged directly in C2M2 by rules governing persistent identifiers) and on the implementation of stable conceptual standards defining meaning and context (so future users can properly explore the data in a way that guarantees meaningful interpretation).

By design, the C2M2 will be amended and extended over time to include additional metadata and relationships, including road-tested community standards, so it can flexibly grow to support any biomedical metadata type and maintain global interoperability. Future work to improve discoverability of CFDE portal resources may, for example, include integrations with harmonizing efforts like schema.org [53] or bioschemas [54]. Technologies like these aim to increase data accessibility via common query interfaces and to improve search engine visibility for indexed resources and datasets; as such things become stable established standards across the research community, they will become candidates for direct incorporation into the C2M2.

The C2M2 supports the optional attachment of persistent, globally resolvable IDs to entity records: these can be used to offer users direct access (via extramural protocols and APIs) to further metadata describing experimental resources of interest, including direct or programmatic download access to files indexed in the portal. A key element of the C2M2 framework, persistent IDs facilitate structured and reliable access to research information housed outside the CFDE portal, which remains stable over time.

### The C2M2 is constantly evolving and expanding

C2M2 modeling decisions are fundamentally intended to strike a balance between 2 goals between which there is a tension that is never fully resolved: ease of data harmonization for submitting DCCs and search effectiveness for researchers using the CFDE portal to find data of interest. Less rigid guardrails around concept harmonization make it easier for DCCs to flexibly create submissions based on information they already have; more tightly enforced unification of descriptive standards makes it easier for users to find what they want.

The process of creating and extending the C2M2 has always had to accommodate a second polarity: balancing the need for future flexibility against maintaining structural consistency over time. Flexibility ensures responsiveness to new research metadata and curation needs; consistency serves as a bulwark against having to refactor existing structures, which is not just expensive but indeed sometimes impossible in principle, as with the case of DCCs whose funding has run out.

Our philosophy of incremental adoption thus applies not only to DCCs building richer C2M2 metadata submissions over time but also to our own process of evolving and expanding the model: throughout, we have grown the model in such a way as to preserve information already built while adding features to accommodate new needs as they arise through our constant dialogues with participating DCCs.

For example: our initial model leaned heavily on data describing files, biosamples, and subjects. As more DCCs with radically different experimental approaches began to participate, it quickly became obvious that a model depending only on these concepts as core entities would leave many of our CF program partners stranded with respect to expressing the most important aspects of their own research. In response—leveraging and expanding the standard scientific vocabularies underpinning C2M2 annotations—we gradually added support for more metadata types describing genes, chemical compounds, phenotypes, proteins, diseases, and more (Table 2 presents a comprehensive list).

We also extended our initial simple concept of C2M2 *collections*—as generalizations of “datasets” that could also include nondata experimental resources—to allow DCCs to directly attach concepts from all supported scientific vocabularies directly to these collections. So whereas anatomical information was initially associated only with biosamples (to clarify sample provenance), anatomy concepts can now be freely associated with arbitrary groups of C2M2 records: sets of data files, for example, can now be bound together into a collection annotated with relevant anatomical information.

Wherever possible, the C2M2 should represent legitimately comparable entities in standard ways without compromising meaning, context, or accuracy. When we encountered the need to weaken precision to preserve search recall, slim maps (see Results, above) were introduced to ensure that the underlying metadata remain broadly findable without sacrificing precision.

C2M2’s mission to faithfully represent similar but distinct packages of important information—taken from multiple independently developed DCC metadata systems—requires ongoing, iterative, case-based design and consensus-driven decision-making, coordinated across multiple research groups. Because model evolution in this context requires long-term planning, testing, and execution, CFDE is committed to handling new metadata that are difficult to integrate and harmonize by adding generalizable, well-defined extensions to C2M2, if possible, and by pruning (at least in the short term) if not. This approach has allowed us to meet the needs of an ever-expanding group of stakeholders and makes C2M2 an ideal framework for other consortia to adopt for their own data curation needs.

## Competing Interests

The authors declare that they have no competing interests.

## Data Availability

Full C2M2 Documentation: https://docs.nih-cfde.org/en/latest/c2m2/draft-C2M2_specification/

C2M2 Supplemental Wiki: https://github.com/nih-cfde/published-documentation/wiki

C2M2 Submission System Documentation: https://docs.nih-cfde.org/en/latest/cfde-submit/docs/

Pubic CFDE files are hosted at OSF: https://osf.io/c8txv/

Selected direct links:

‐ Submission preparation script: https://osf.io/bq6k9/
‐ CFDE defined controlled vocabularies: https://osf.io/m3a85/
‐ C2M2 master JSON schema: https://osf.io/c63aw/

**Portal code:**

CFDE specific Deriva fork: https://github.com/nih-cfde/cfde-deriva

- Operating system: Linux
- Programming language: Python with some SQL
- Other requirements: Python 3.6+, bdbag 1.6.4+, frictionless 4.0.3+, deriva 1.5.0+

Portal UI customization: https://github.com/nih-cfde/dashboard

- Operating system(s): Platform independent
- Programming language: JavaScript, HTML, CSS
- Other requirements: A web server (e.g., Apache HTTP Server)
- License: Creative Commons CC0

Portal UI customization API: https://github.com/nih-cfde/dashboard-api

- Operating system(s): Platform independent
- Programming language: Python 3.6+
- Other requirements: A web server (e.g., Apache HTTP Server); Flask; cfde-deriva and deriva-core
- License: Creative Commons CC0

**Submission code:**

CFDE specific Globus flow fork: https://github.com/nih-cfde/cfde-submit

- Operating systems: Platform independent. Unit tests run on Linux, Windows, and Mac through GitHub actions
- Programming language: Python
- Other requirements: Git, Python 3.6+
- License: Apache 2.0

CFDE specific Globus action provider fork: https://github.com/nih-cfde/deriva-action-provider

- Operating system: Linux
- Programming language: Python
- Other requirements: None
- License: Apache 2.0

Archival copies of the GitHub repositories are also available via the GigaScience database GigaDB [55].

## Abbreviations

C2M2: Crosscut Metadata Model; CF: Common Fund; CFDE: Common Fund Data Ecosystem; CFDE-CC: Common Fund Data Ecosystem Coordinating Center; CURIE: Compact Uniform Resource Identifier; DCC: Data Coordination Center; ER: entity-relationship; ExRNA: Extracellular RNA; FAIR: findability, accessibility, interoperability, and reusability; GMKF: Gabriella Miller Kids First Pediatric Research Program; GTEx: Genotype-Tissue Expression; HMP: Human Microbiome Project; HuBMAP: Human BioMolecular Atlas Project; IDG: Illuminating the Druggable Genome; LINCS: Library of Integrated Network-Based Cellular Signatures; NIH: National Institutes of Health; OWG: Ontology Working Group; QTL: quantitative trait locus; RFC: Requests for Comment; RNA-seq: RNA sequencing; TSV: tab-separated value file; UBERON: Uber-anatomy Ontology.

## Authors’ Contributions

ALC Conceptualization, Formal Analysis, Funding acquisition, Investigation, Methodology, Project administration, Supervision, Validation, Visualization, Writing – original draft, Writing – review & editing AB Conceptualization, Data curation, Methodology, Project administration, Software, Validation, Writing – original draft, Writing – review & editing, C2M2 development lead K Czajkowski Conceptualization, Formal Analysis, Software, Visualization JA Data curation SC Methodology, Validation, Writing – review & editing RC Funding acquisition, Project administration, Supervision K Chard Software, Supervision, Writing – review & editing DJBC Data curation, Investigation, Software, Writing – review & editing JC Software, Visualization HC Data curation, Validation MD Conceptualization, Software VF Software, Supervision, Visualization MG Conceptualization, Data curation, Methodology, Project administration, Supervision, Writing – review & editing AG Conceptualization, Writing – review & editing, Provided clinical context from user point-of-view and review of the manuscript RMH Project administration, Validation, Writing – review & editing TKH Data curation, Data Harmonization OI Data curation MJ Methodology, Resources, Software JL Project administration, Validation EK Data curation MCWL Methodology, Validation, Writing – review & editing RLL Project administration, Software, Supervision, Writing – review & editing AAM Conceptualization, Funding acquisition, Project administration, Supervision MM Investigation JBM Data curation, Writing – review & editing SN Data curation, Validation, Data harmonization RR Software CR Methodology, Validation PR Conceptualization, Methodology, Writing – review & editing MS Software, Visualization RES Investigation, Software, Writing – review & editing HT Software, Supervision AW Project administration CW Writing – review & editing KWSusanna-Assunta Sansone Conceptualization, Writing – review & editing AM Investigation, Methodology, Supervision, Writing – review & editing RW Conceptualization, Project administration, Software, Validation IF Conceptualization, Funding acquisition, Methodology, Project administration, Software, Supervision, Writing – review & editing CK Conceptualization, Data curation, Methodology, Software CTB Conceptualization, Funding acquisition, Project administration, Supervision, Writing – original draft, Writing – review & editing OW Conceptualization, Funding acquisition, Project administration, Supervision, Writing – original draft, Writing – review & editing All authors reviewed the final manuscript.

## Funding

NIH Common Fund OT3OD025459-01 for the CFDE Coordinating Center.

## Supplementary Material

giac105_GIGA-D-22-00120_Original_Submission

giac105_GIGA-D-22-00120_Revision_1

giac105_Response_to_Reviewer_Comments_Original_Submission

giac105_Reviewer_1_Report_Original_Submission J. Harry Caufield, Ph.D. -- 5/26/2022 Reviewed

giac105_Reviewer_2_Report_Original_Submission Carole Goble -- 6/27/2022 Reviewed

giac105_Supplemental_Files

## Supplementary Data

C2M2 Persistent IDs, Full entity-relationship (ER) diagram describing C2M2
