# Supplementary material for: Making Common Fund data more findable: catalyzing a data ecosystem
Source: Gigascience. 2022 Nov 21;11:giac105. doi: 10.1093/gigascience/giac105 (PMC9677336; doi:10.1093/gigascience/giac105)
Supplement: giac105_Response_to_Reviewer_Comments_Original_Submission [file giac105_response_to_reviewer_comments_original_submission.pdf]

Author's Response To Reviewer Comments

Close

Reviewer #1:

Major issues:
1. As noted on page 11, "All C2M2 controlled vocabulary annotations are optional". Data producers will use terms outside the controlled vocabulary as needed, and are unlikely to consult any CFDE working groups in every instance. Is there some automated system for term normalization in place? How will data producers be encouraged to preferentially use controlled terms? Are they warned during submission, as noted on page 22 regarding data contents?
(Note: this section now begins at the bottom of page 14)
Submitters may or may not submit controlled vocabulary terms at their own discretion, but by "optional" we mean only that these fields can be left blank, not that submitters can substitute their own terms: we don't accept any terms outside supported vocabularies. We also have recently integrated a report page that summarizes inter-entity connectivity of their submission and suggests good next steps for improvement. We have clarified the indicated section to better convey our meaning:
"C2M2 currently provides a variety of (optional) fields and tables through which controlled (standardized, curated) scientific vocabulary terms can be attached to C2M2 collections and entities."
We have updated the legend for Figure 3 (the review page that includes a snapshot of the connectivity report)
We have clarified the language about the connectivity report in the Results section ("The CFDE provides robust data ingest and validation for data packages," top of p. 20)
We have also clarified related language in the Discussion section ("The C2M2 is designed to support incremental adoption," top of p. 30)

Minor comments:
2. The first example of the mismatch between user expectations and actual results of searching for Common Fund program data is very illustrative. I appreciate how it notes that even instances like matching Dr. Phil Blood's name in a search can complicate Findability.
Thank you!
3. The abstract could include some brief description of the broader relevance and impact of the metadata model, including its potential for use outside the CFDE.
We have added "This Crosscut Metadata Model (C2M2) supports the wide variety of data types and metadata terms used by individual DCCs and can readily describe nearly all forms of biomedical research data" to the abstract.
4. On page 5, the sentence "Thus, a researcher interested in combining data across CF programs is faced with not only a huge volume, richness, and complexity of data, but also a wide variety, richness, and complexity of data access systems with their own vocabularies, file types, and data structures" feels somewhat redundant and could benefit from some editing.
Rewrite: "Researchers interested in combining data across CF programs must therefore not only find the datasets, but then harmonize across idiosyncratic vocabularies, file types, data structures and processing methods."
5. The structure of Figure 1 (or should this be Table 1?) is confusing. The general idea is clear - metadata types, properties, and formats are inconsistent across projects - but the two-column format presents issues with direct comparison.
We have reconfigured this figure into a split table, making term usage at DCCs more easily visually comparable. DCCs are now columns, with key/value sets categorized by search concept. The main text has also been updated to discuss an explicit comparison.
6. It is interesting that, among all values presented in Fig. 1, just one includes a CURIE (HMP's ENVO:02000020). This may be worth further comment as it is striking that few of these projects have adopted unique identifiers within their metadata schemata.
This is a great point. We have highlighted in the Introduction the fact that each DCC largely created their own models, datatypes, storage, etc.; we've Rewritten the Results subsection "Common Fund data cannot be found by uniform Internet search terms" to discuss it as well, and we now more thoroughly address this in the new section summarizing our findings from the listening tour.

7. Slightly more detail regarding the interviews with Common Fund programs would be helpful for understanding how these interactions contributed to the process. Were interviews primarily with PIs? Were several prominent issues repeatedly discussed in the context of multiple projects?
We've added substantial information to the "A listening tour identified obstacles to interoperation" section to address this question. Also highlighted is the fact that full reports of these visits have been published on figshare and are available to anyone who is interested in more.

8. Is the C2M2 master JSON schema publicly accessible?
It is! It is linked from our documentation, but we've also added direct links to our public files in the Data Availability section.
9. Some redundancy is present between the first and second paragraphs under the heading "Entities and associations are key structural features of the C2M2" - e.g., core entities and container entities are both described twice.
The "core entity" concept has frankly been superseded by a more flexible philosophy; we've restructured this section both to remove the observed redundancy and to clarify our current context and approach.
10. In Figure 2, some lines connecting tables are very close to the edge of the figure borders and are difficult to see as a result.
Image margins have been increased.
11. Is there a mechanism for dealing with obsolete terms as the ontologies contributing to the controlled vocabulary change? In the event that the NCBI Taxonomy renames a genus, for example, how will CFDE metadata change (if at all)?
This is somewhat context dependent. For datasets that are still actively maintained by their DCC, we would expect the DCC to update their metadata accordingly. If the dataset has been transitioned to CFDE-CC ownership and is funded to be periodically refreshed, we would update the terms as needed. If the dataset is no longer updated, it will retain its original terms, but the portal user interface will still be able to display obsolete terms. We have also refactored the end of the "The C2M2 integrates standardized vocabularies" subsection to address this.:
"We also note that the same infrastructure that facilitates this enhanced synchronization with C2M2-supported vocabularies, allowing the incorporation of provisional terms into the C2M2, can also be used to maintain obsolete terms in cases where the original dataset cannot be updated but subsequent curation of the relevant vocabulary has removed them."

Reviewer #2:

The process of working with the Centers is interesting as is the choice of three core entities. Some more discussion on why these three and only these three would be appreciated.
These were the three entities (files, subjects and biospecimens) that all the initial DCCs had mappable concepts for, and so initially formed the 'base' of the model which we expected nearly all data providers would be able to fill. As more DCCs came onboard these assumptions proved too inflexible; all references to "core entities" have been removed, and an entirely new summary of this phenomenon has been added to the "The C2M2 is constantly evolving and expanding" subsection of the Discussion:
"...our initial model leaned heavily on data describing files, biosamples and subjects. As more DCCs with radically different experimental approaches began to participate, it quickly became obvious that a model depending only on these concepts as core entities would leave many of our CF Program partners stranded with respect to expressing the most important aspects of their own research. In response – leveraging and expanding the standard scientific vocabularies underpinning C2M2 annotations – we gradually added support for more metadata types describing genes, chemical compounds, phenotypes, proteins, diseases and more (Table 2 presents a comprehensive list)."

The ingest pipeline and process is not so clear.
- It seems that each Center is required to map its datasets to the current C2M2 model in 48 TSV files, in a data package that is then uploaded to the catalogue and ingested into the portal's database. Is the data package a complete reupload each time or is the data package additive? There are hints in the text that it is a replacement each time.
It is replaced each time: we have rewritten the relevant parts of "The CFDE provides robust data ingest and validation for data packages" – please see specifically the end of that section on p. 20:
"All CFDE metadata releases include exactly one submission from each participating DCC: on every (quarterly) release date, the newest approved submission from each DCC becomes findable, browsable and searchable in the public CFDE portal. If a DCC does not submit and approve an updated C2M2 submission between releases, their most recent public submission remains accessible without

modification; if a new submission is approved, at the next release it will completely replace that DCC's previously-available data. Incremental (partial) data updates are not supported: they are generally error-prone and create increasing difficulty over time for DCC curators trying to maintain guarantees of stable data provenance and reusability."

- What is the cost and complexity of this mapping and upload borne by the Centers? Any insights would be valuable. Is and tooling provide to help beyond the documentation?
Initial mapping can be difficult, but we encourage Centers to start small and build up complexity over time. The NIH also provides funding for Centers to work with us. We do provide additional tooling as well. Tooling is now listed explicitly in the data availability section. We have also renamed "Use of the C2M2 is supported by extensive documentation" to "Data submission is supported and subsidized by the CFDE ecosystem" and substantially rewritten it to more clearly describe this support

- Figure 5 could be improved to include the data that flows between the steps, and the actors. Could Figure 3 and 5 be merged?
That's a great idea, thank you! These two figures have now been merged and the description has been updated to match.

- If the datasets are reuploaded afresh each cycle, how are between-release analytics managed? By the use of the PIDs? Are there any restrictions on what cannot be changed between releases?
Currently there are no restrictions on what can be changed. In the private portion of the portal where DCCs review their data prior to publication, they also have access to all previous data packages that were part of a release; these can be used to assess changes. Because the model – and how DCCs harmonized their data to it – evolved substantially over the first year of use, we do not yet have analytics in place for individual entities (file, biosample, etc,), but instead do bulk analysis of CV term usage and how those shift over time. These basic analyses are available in our public Working Group google drive folder here:
https://drive.google.com/drive/u/0/folders/1ghfrUZNidNlw891BgM1bKZB5NX7ByRuL

- As the datasets can be incrementally improved with each release, are there any trends between releases that indicate changes in metadata enrichment - On page 18 you state that "DCCs get better at using the C2M2"
In general we see that with each successive submission each DCC submits both more data and more connected data. We've added this to the paragraph indicated, and have also added relevant text to the new section for Reviewer #1 (point 1) discussing controlled vocabularies.

- The data package needs clearer description: relationship between the TSV files, the Frictionless Data JSON and BDBag is of interest to many in the community and warrant a more thorough discussion.
We have completely rewritten several relevant sections:
"A C2M2 data package consists of 48 TSV files (as of 4/4/2022) populated with interrelated metadata about DCC data assets. Precise formatting requirements for data packages are specified by a JSON Schema document that describes the entire C2M2. The C2M2 JSON Schema document is itself structured according to the "Data Package" meta-specification published by the Frictionless Data group [36]: implementing this structural standard enables DCCs and the CFDE to leverage an existing ecosystem of validation tools. The C2M2 specification defines foreign-key relationships between metadata fields (TSV columns), rules governing missing data, details governing required content types and formats for individual fields, and other constraints. These architectural rules ensure the internal structural integrity of each individual C2M2 submission. Most importantly, they also serve to guarantee and maintain a unified, harmonized C2M2 database underpinning the CFDE portal, automatically enabling compatibility and interoperability across multiple federated submissions received from different DCCs at different times." (p. 17)
"Each TSV in a C2M2 submission is a plain-text file representing a tabular data matrix, with the first (header) line listing tab-separated column names, and any subsequent lines representing table rows (records) with tab-separated column values (fields). TSVs must conform to all formatting and relational constraints in the C2M2 JSON Schema document [37]. Any blank table is submitted as a TSV containing just the header line – requiring inclusion of these stub TSVs helps differentiate between intentional and accidental data omission." (& ff, p. 18)
"The [cfde-submit] tool scans a directory of C2M2 TSVs, validates their contents against the C2M2 JSON Schema, and builds a BDBag file [41] containing the TSVs, a copy of the schema, and some minimal provenance metadata. The tool then securely uploads the BDBag to a dedicated DCC-specific Globus [42] endpoint provided by the CFDE-CC during onboarding; only explicitly authorized DCC users can submit to their DCC's endpoint." (& ff., p. 19)

The portal
- Why were these three basic kinds of search chosen? Were there user stories collected from the listening tour?
We do have user stories for these searches, but also practical limitations of the available data. We have added detailed illustrative examples to elaborate on each of the three search types.

- It would be helpful if there were some indications of the use of the catalogue by users rather than just the ingest and publishing pipeline.
We agree! As the portal had its first public release in February of 2022, we are still working to publicize and build a user base. We hope that this publication will help with that effort. We've added some summary public release data:
"As of our first public release in February 2022, the CFDE makes 3,041,978 files, 1,749,145 biosamples, and 34,375 subjects from eleven Programs searchable in a single harmonized interface." (pp. 23-24)
Page 5 the arguments are made that reusing common fund data for cross-cutting analysis is challenging and requires the hiring of dedicated bioinformaticians ("at considerable cost of NIH"). How does making the datasets available through a catalogue relieve the burden of skilled bioinformaticians to analyse data? The data still needs to be processed. Hasn't the burden just shifted to the Centers to prepare the TSV files for the ingest pipeline?
The challenges listed in the prior sentence (working with large files, accessing data in the cloud, harmonization, and data transformation) are all steps that occur prior to what most people consider 'analysis', and are the steps that we were referring to here – specifically in the context of bioinformaticians using the CFDE portal to find datasets, as opposed to DCC staff creating C2M2 submissions (which still clearly requires dedicated expert work). We have updated this section to clarify:
"Comparing data across programs is particularly challenging: each program's data portal provides a curated experience of analyzed data that usually does not support comparison with data from other sources. Moreover, many data can only be meaningfully compared to other data processed by the same methods, and because each Common Fund program operates independently, data are stored, labeled, analyzed, curated and maintained in incompatible ways. Researchers interested in combining data across CF programs must therefore not only find the datasets, but then harmonize across idiosyncratic vocabularies, file types, data structures and processing methods. Reusing Common Fund data for new cross-cutting scientific analyses requires expertise in working with large files, accessing data in the cloud, harmonization, and data transformation -- all before any actual analysis can begin. Each of these processes typically individually represents a significant challenge for biomedical researchers and clinicians, motivating labs to hire dedicated bioinformaticians (at considerable cost) to manage them. Confronting all of these data-munging operations – necessary before researchers can even begin integrative analysis – often proves prohibitive, truncating or outright eliminating potential investigations." (p. 5)
However, we also do believe this work will impact analysis. There are reliable tools for all the science processing: calling variants, alignments, gene discovery, that are easily automatable. As a bioinformatician, who works with bioinformaticians, and has trained hundreds of fledgling bioinformaticians, I have found that the vast majority of the time bioinformaticians spend on data analysis is not in running the pipeline, but in finding, and cleaning, and munging and harmonizing the data themselves:

http://biocomicals.blogspot.com/2011/05/thats-what-bioinformaticians-do.html#.TpRFPJQEyMU.twitter
A portal that improves FAIRness by allowing users to find data quickly and be pointed directly at how to download it saves a huge amount of time. But just as importantly we are taking the burden of figuring out how to best harmonize the data off of the plate of each bioinformatician who needs it, and centralizing that burden to the DCCs. This both saves time and energy on the part of the bioinformaticians, and improves the quality of the data. The metadata submitted to the CFDE portal is formatted into the C2M2 by the data owners, who are by definition most qualified to understand what their terms mean and map to. By having all participating DCCs match their data to the same model, we are de facto harmonizing those data points. Rather than every paper who co-analyzes both GTEx and Kids First data needing to establish their own (idiosyncratic) harmonization, they can leverage the harmonization that has already been done by the DCCs who have the benefit of their own deep knowledge of the data, plus the ability to directly interact with other CFDE DCCs to agree in advance on how terms should be used (via e.g. the ontology working group) and on what standard vocabularies everyone should adopt (via e.g. the anatomy, gene, variant, and clinical metadata working groups). This will result in analyses (by researchers using the CFDE portal) that are both higher quality, because we can be sure that the mapping was as good as is possible, more comparable to one another, and more repeatable, because as everyone who reuses this data will have begun with the same mapping scheme.

We have added a new section to the Discussion covering this topic directly: “The C2M2 and the CFDE portal improve FAIRness”

Page 5 claims that the sociotechnical framework of the CFDE is a self-sustaining community. How? Working groups have been established but to what extent are these managed by the community and not the dedicated action of developing the portal? What is the sustainability of the portal?
The position of the CFDE Coordination Center is, and always has been, that if we do our jobs correctly, those jobs will cease to exist, and we have worked to set up the CFDE in such a way that we will ultimately be minimally needed. The CFDE working groups are entirely community-based. They are suggested, chaired, and crewed entirely by DCC members (CFDE staff and NIH program officers also attend), and part of the sustainability is tech/community/understanding based: everyone at least uses a common submission pipeline, and over time C2M2 has converged on shared principles. CFDE staff act only to mediate and to provide and logistics support: we introduce DCCs to one another, we manage calendars, we make zoom meetings and distribute recordings. If the Coordination Center was de-funded tomorrow, our role in working groups could in most cases easily be replaced by any decent secretary. We have added language about this to the new section describing the listening tour, as well as adding the following to the “Data submission is supported and subsidized by the CFDE ecosystem” subsection in the Discussion:
“The CFDE as a whole also offers substantial social support, decentralized with minimal reliance on the CFDE Coordination Center. Our position at the CFDE-CC is that if we do our jobs correctly, those jobs will cease to exist. Our goal is to build a self-sustaining community that actively works towards interoperability as each new DCC begins its funding lifecycle, so that integration challenges documented on our listening tour become relics of the past. As such, the CFDE-CC provides both technical infrastructure and administrative logistics (calendering, meeting space, C2M2, portal, etc) to support DCCs coming together to make decisions and harmonize data. CFDE working groups guiding critical aspects of the ecosystem are proposed, chaired, run, attended, and dissolved by the DCCs themselves. These groups have allowed the community to flexibly focus on short-term goals, as well as establishing forward-leaning conversations about emerging challenges and new directions, driven entirely by the DCCs.” (pp. 27-28)
The easy expansion of the C2M2 seems to depend on two things: the incorporation of domain specific vocabularies and the cycle of ingest-releases at time points. Does this latter point constitute expansion that is easy? This would require each data center to adapt to the new table templates.
While the C2M2 is flexible and updates frequently, we have to date managed to ensure that updates happen only by extension, and not modification or refactoring of existing tables. Our ingest system can therefore easily integrate older submissions (lacking newer fields and tables) by assuming that metadata corresponding to the newer additions has not yet been provided. DCCs that stop updating their data would thus just remain searchable by their current data, and have blanks (nulls, data not present) for any new features. We’ve added a discussion of this phenomenon and its motivation to the “The C2M2 is constantly evolving and expanding” subsection:
“The process of creating and extending the C2M2 has always had to accommodate a second polarity: balancing the need for future flexibility against maintaining structural consistency over time. Flexibility insures responsiveness to new research metadata and curation needs; consistency serves as a bulwark against having to refactor existing structures, which is not just expensive but indeed sometimes impossible in principle, as with the case of DCCs whose funding has run out.

Our philosophy of incremental adoption thus applies not only to DCCs building richer C2M2 metadata submissions over time, but also to our own process of evolving and expanding the model: throughout, we have grown the model in such a way as to preserve information already built while adding features to accommodate new needs as they arise through our constant dialogues with participating DCCs.” (p. 33)

Page 9 Containers are mentioned but it is not clear what the difference is between a container and a collection. Containers do not seem to appear again in the browsing.
We have rewritten this entire section to clarify; relevant language now includes
“We refer to C2M2 project and collection entities as containers: named sets or groups containing particular C2M2 files, biosamples and/or subjects. Containers were designed not only to allow DCCs to explicitly and flexibly group related entity records into named sets, but also to associate these groups with well-defined scientific concepts (described in the next section).” (pp. 12-13)
Page 18 the visibility of Biosamples changing over time in Figure 4 wasn't so clear to me
This was a typo; we meant to refer to what was originally submitted as Figure 6, which has providentially since been renamed to Figure 4, hopefully taking care of this bit.

Close
