# Supplementary material for: Making Common Fund data more findable: catalyzing a data ecosystem
Source: Gigascience. 2022 Nov 21;11:giac105. doi: 10.1093/gigascience/giac105 (PMC9677336; doi:10.1093/gigascience/giac105)
Supplement: giac105_Reviewer_1_Report_Original_Submission — J. Harry Caufield, Ph.D. -- 5/26/2022 Reviewed [file giac105_reviewer_1_report_original_submission.pdf]

Reviewer Report

Title: Making Common Fund data more findable: Catalyzing a Data Ecosystem

Version: Original Submission **Date:** 5/26/2022

Reviewer name: J. Harry Caufield, Ph.D.

Reviewer Comments to Author:

This manuscript by Charbonneau et al. details efforts to address challenges in enhancing the value of metadata among projects in the NIH's Common Fund Data Ecosystem. They specifically detail how a new metadata model was developed and deployed to unify data properties across projects. Assembling such a model is a major accomplishment and a necessary step in promoting data reuse. Applying the model is another commendable achievement. The manuscript text undersells the value of these efforts. How has the value of data in the CFDE improved due to implementation of a unified metadata model and new infrastructure? The authors clearly delineate the challenges in searching CFDE data; these issues frequently appear in efforts toward improving biomedical data FAIRness and are directly relevant to the core challenges identified by Wilkinson et al. (2016) in their FAIR guiding principles. Much more emphasis could be placed on the overall impact of a consistent metadata model, whether within the CFDE alone or in the broader realm of bio-data management.

Major issues:

1. As noted on page 11, "All C2M2 controlled vocabulary annotations are optional". Data producers will use terms outside the controlled vocabulary as needed, and are unlikely to consult any CFDE working groups in every instance. Is there some automated system for term normalization in place? How will data producers be encouraged to preferentially use controlled terms? Are they warned during submission, as noted on page 22 regarding data contents?

Minor comments:

2. The first example of the mismatch between user expectations and actual results of searching for Common Fund program data is very illustrative. I appreciate how it notes that even instances like matching Dr. Phil Blood's name in a search can complicate Findability.
3. The abstract could include some brief description of the broader relevance and impact of the metadata model, including its potential for use outside the CFDE.
4. On page 5, the sentence "Thus, a researcher interested in combining data across CF programs is faced with not only a huge volume, richness, and complexity of data, but also a wide variety, richness, and complexity of data access systems with their own vocabularies, file types, and data structures" feels somewhat redundant and could benefit from some editing.
5. The structure of Figure 1 (or should this be Table 1?) is confusing. The general idea is clear - metadata types, properties, and formats are inconsistent across projects - but the two-column format presents issues with direct comparison.
6. It is interesting that, among all values presented in Fig. 1, just one includes a CURIE (HMP's ENVO:02000020). This may be worth further comment as it is striking that few of these projects have adopted unique identifiers within their metadata schemata.

7. Slightly more detail regarding the interviews with Common Fund programs would be helpful for understanding how these interactions contributed to the process. Were interviews primarily with PIs? Were several prominent issues repeatedly discussed in the context of multiple projects?
8. Is the C2M2 master JSON schema publicly accessible?
9. Some redundancy is present between the first and second paragraphs under the heading "Entities and associations are key structural features of the C2M2" - e.g., core entities and container entities are both described twice.
10. In Figure 2, some lines connecting tables are very close to the edge of the figure borders and are difficult to see as a result.
11. Is there a mechanism for dealing with obsolete terms as the ontologies contributing to the controlled vocabulary change? In the even that the NCBI Taxonomy renames a genus, for example, how will CFDE metadata change (if at all)?

Methods

Are the methods appropriate to the aims of the study, are they well described, and are necessary controls included? Choose an item.

Conclusions

Are the conclusions adequately supported by the data shown? Choose an item.

Reporting Standards

Does the manuscript adhere to the journal's guidelines on minimum standards of reporting? Choose an item.

Choose an item.

Statistics

Are you able to assess all statistics in the manuscript, including the appropriateness of statistical tests used? Choose an item.

Quality of Written English

Please indicate the quality of language in the manuscript: Choose an item.

Declaration of Competing Interests

Please complete a declaration of competing interests, considering the following questions:

- Have you in the past five years received reimbursements, fees, funding, or salary from an organisation that may in any way gain or lose financially from the publication of this manuscript, either now or in the future?
- Do you hold any stocks or shares in an organisation that may in any way gain or lose financially from the publication of this manuscript, either now or in the future?
- Do you hold or are you currently applying for any patents relating to the content of the manuscript?
- Have you received reimbursements, fees, funding, or salary from an organization that holds or has applied for patents relating to the content of the manuscript?
- Do you have any other financial competing interests?
- Do you have any non-financial competing interests in relation to this paper?

If you can answer no to all of the above, write 'I declare that I have no competing interests' below. If your reply is yes to any, please give details below.

I declare that I have no competing interests.

I agree to the open peer review policy of the journal. I understand that my name will be included on my report to the authors and, if the manuscript is accepted for publication, my named report including any attachments I upload will be posted on the website along with the authors' responses. I agree for my report to be made available under an Open Access Creative Commons CC-BY license (http://creativecommons.org/licenses/by/4.0/). I understand that any comments which I do not wish to be included in my named report can be included as confidential comments to the editors, which will not be published.

Choose an item.

To further support our reviewers, we have joined with Publons, where you can gain additional credit to further highlight your hard work (see: https://publons.com/journal/530/gigascience). On publication of this paper, your review will be automatically added to Publons, you can then choose whether or not to claim your Publons credit. I understand this statement.

Yes Choose an item.
