# Supplementary material for: Making Common Fund data more findable: catalyzing a data ecosystem
Source: Gigascience. 2022 Nov 21;11:giac105. doi: 10.1093/gigascience/giac105 (PMC9677336; doi:10.1093/gigascience/giac105)
Supplement: giac105_Reviewer_2_Report_Original_Submission — Carole Goble -- 6/27/2022 Reviewed [file giac105_reviewer_2_report_original_submission.pdf]

Reviewer Report

Title: Making Common Fund data more findable: Catalyzing a Data Ecosystem

Version: Original Submission **Date:** 6/27/2022

Reviewer name: Carole Goble

Reviewer Comments to Author:

The article is a very useful contribution to the growing number of metadata models and data catalogues in the life science data ecosystem. The recent NIH mandates in data sharing emphasize the need for findability of datasets, and the need to operate within a federation and ecosystem recognises the reality of independent data centers and legacy data collections.
The paper states the context of the CFDE well, setting up the need for a centralized portal capable of ingesting, indexing, search and supporting cross dataset comparisons of dataset from different, independent data centers without the need for those centers to move, reformat or rehost their data. This is a common pattern that many data infrastructure providers will recognise.
The incremental approach that supports minimal uploads and respects local DOI implementation is a pragmatic approach that has made onboarding the data centers feasible, I suspect.
The insight that mapping to common ontologies does not actually lead to harmonised dataset and nor does it support search is a useful lesson that resonates and is useful to reiterate (although it is already well known).
Given the approach is tabular, Frictionless data makes sense.
The process of working with the Centers is interesting as is the choice of three core entities. Some more discussion on why these three and only these three would be appreciated.
The ingest pipeline and process is not so clear.
- It seems that each Center is required to map its datasets to the current C2M2 model in 48 TSV files, in a data package that is then uploaded to the catalogue and ingested into the portal's database. Is the data package a complete reupload each time or is the data package additive? There are hints in the text that it is a replacement each time.
- What is the cost and complexity of this mapping and upload borne by the Centers? Any insights would be valuable. Is and tooling provide to help beyond the documentation?
- Figure 5 could be improved to include the data that flows between the steps, and the actors. Could Figure 3 and 5 be merged?
- If the datasets are reuploaded afresh each cycle, how are between-release analytics managed? By the use of the PIDs? Are there any restrictions on what cannot be changed between releases?
- As the datasets can be incrementally improved with each release, are there any trends between releases that indicate changes in metadata enrichment - On page 18 you state that "DCCs get better at using the C2M2"
- The data package needs clearer description: relationship between the TSV files, the Frictionless Data JSON and BDBag is of interest to many in the community and warrant a more thorough discussion.
The portal

- Why were these three basic kinds of search chosen? Were there user stories collected from the listening tour?
- It would be helpful if there were some indications of the use of the catalogue by users rather than just the ingest and publishing pipeline.

Page 5 the arguments are made that reusing common fund data for cross-cutting analysis is challenging and requires the hiring of dedicated bioinformaticians ("at considerable cost of NIH"). How does making the datasets available through a catalogue relieve the burden of skilled bioinformaticians to analyse data? The data still needs to be processed. Hasn't the burden just shifted to the Centers to prepare the TSV files for the ingest pipeline?

Page 5 claims that the sociotechnical framework of the CFDE is a self-sustaining community. How? Working groups have been established but to what extent are these managed by the community and not the dedicated action of developing the portal? What is the sustainability of the portal?

The easy expansion of the C2M2 seems to depend on two things: the incorporation of domain specific vocabularies and the cycle of ingest-releases at time points. Does this latter point constitute expansion that is easy? This would require each data center to adapt to the new table templates.

Page 9 Containers are mentioned but it is not clear what the difference is between a container and a collection. Containers do not seem to appear again in the browsing.

Page 18 the visibility of Biosamples changing over time in Figure 4 wasn't so clear to me

Methods

Are the methods appropriate to the aims of the study, are they well described, and are necessary controls included? Choose an item.

Conclusions

Are the conclusions adequately supported by the data shown? Choose an item.

Reporting Standards

Does the manuscript adhere to the journal's guidelines on [minimum standards of reporting?] Choose an item.

Choose an item.

Statistics

Are you able to assess all statistics in the manuscript, including the appropriateness of statistical tests used? Choose an item.

Quality of Written English

Please indicate the quality of language in the manuscript: Choose an item.

Declaration of Competing Interests

Please complete a declaration of competing interests, considering the following questions:

- Have you in the past five years received reimbursements, fees, funding, or salary from an organisation that may in any way gain or lose financially from the publication of this manuscript, either now or in the future?
- Do you hold any stocks or shares in an organisation that may in any way gain or lose financially from the publication of this manuscript, either now or in the future?
- Do you hold or are you currently applying for any patents relating to the content of the manuscript?
- Have you received reimbursements, fees, funding, or salary from an organization that holds or has applied for patents relating to the content of the manuscript?
- Do you have any other financial competing interests?
- Do you have any non-financial competing interests in relation to this paper?

If you can answer no to all of the above, write 'I declare that I have no competing interests' below. If your reply is yes to any, please give details below.

'I declare that I have no competing interests'

I agree to the open peer review policy of the journal. I understand that my name will be included on my report to the authors and, if the manuscript is accepted for publication, my named report including any attachments I upload will be posted on the website along with the authors' responses. I agree for my report to be made available under an Open Access Creative Commons CC-BY license (http://creativecommons.org/licenses/by/4.0/). I understand that any comments which I do not wish to be included in my named report can be included as confidential comments to the editors, which will not be published.

Choose an item.

To further support our reviewers, we have joined with Publons, where you can gain additional credit to further highlight your hard work (see: https://publons.com/journal/530/gigascience). On publication of this paper, your review will be automatically added to Publons, you can then choose whether or not to claim your Publons credit. I understand this statement.

Yes Choose an item.
